# Long‐lived *Plasmodium falciparum* specific memory B cells in naturally exposed Swedish travelers

**DOI:** 10.1002/eji.201343630

**Published:** 2013-08-29

**Authors:** Francis M. Ndungu, Klara Lundblom, Josea Rono, Joseph Illingworth, Sara Eriksson, Anna Färnert

**Affiliations:** ^1^ Centre for Geographical Medicine Research (Coast)Kenya Medical Research InstituteKilifiKenya; ^2^ Infectious Diseases UnitDepartment of Medicine SolnaKarolinska InstituteStockholmSweden; ^3^ Department of Infectious DiseasesKarolinska University HospitalStockholmSweden; ^4^ Centres for Clinical Vaccinology and Tropical MedicineNuffield Department of MedicineUniversity of OxfordOxfordUK

**Keywords:** Antibodies, Longevity, Malaria, Memory B cells, *Plasmodium falciparum*

## Abstract

Antibodies (Abs) are critical for immunity to malaria. However, *Plasmodium falciparum* specific Abs decline rapidly in absence of reinfection, suggesting impaired immunological memory. This study determines whether residents of Sweden that were treated for malaria following international travel maintained long‐lasting malaria‐specific Abs and memory B cells (MBCs). We compared levels of malaria‐specific Abs and MBCs between 47 travelers who had been admitted with malaria at the Karolinska University Hospital between 1 and 16 years previously, eight malaria‐naïve adult Swedes without histories of travel, and 14 malaria‐immune adult Kenyans. *Plasmodium falciparum*‐lysate‐specific Ab levels were above naïve control levels in 30% of the travelers, whereas AMA‐1, merozoite surface protein‐1_42_, and merozoite surface protein‐3‐specific Ab levels were similar. In contrast, 78% of travelers had IgG‐MBCs specific for at least one malaria antigen (59, 45, and 28% for apical merozoite antigen‐1, merozoite surface protein‐1, and merozoite surface protein‐3, respectively) suggesting that malaria‐specific MBCs are maintained for longer than the cognate serum Abs in the absence of re‐exposure to parasites. Five travelers maintained malaria antigen‐specific MBC responses for up to 16 years since the diagnosis of the index episode (and had not traveled to malaria‐endemic regions in the intervening time). Thus *P. falciparum* can induce long‐lasting MBCs, maintained for up to 16 years without reexposure.

## Introduction

There is long‐standing evidence that naturally acquired immunity to the erythrocytic stages of malaria, which limits morbidity and mortality in older children and adults, is strongly dependent on antibody (Ab; reviewed in 1). Since naturally acquired immunity to malaria is acquired following repeated exposure to parasites, it may diminish with the recently reported declining *Plasmodium falciparum* transmission in parts of Africa, rendering formerly immune individuals or susceptible populations again 4. If this happens, the public health gains from sustained malaria control programs 7 would be threatened by the resultant increase in susceptibility of largely nonimmune populations if malaria transmission was resurgent.

Although immunoepidemiological studies conducted in different geographical locations have not yet produced a clear picture of which antigen targets are the most important with regards to naturally acquired immunity to malaria, there is a general consensus that Abs are critical for protection 8. However, malaria‐specific Abs may be short‐lived in young children 11, or long‐lived in older individuals 16, suggesting that B‐cell memory, as encoded in memory B cells (MBCs) and plasma cells, is dysfunctional in early life. Additionally, persistent *P. falciparum* infections drive the expansion of atypical MBCs 18, an MBC phenotype that was previously shown to be exhausted in viremic HIV infections 21. Although atypical MBCs may not be exhausted in the context of persistent *P. falciparum* infections 23, the durability and function of B‐cell memory in malaria remains an interesting area of research.

Unlike serum Abs that can be short‐lived in the absence of persistent antigen, human MBCs are generally long‐lived 24. For example, anti‐vaccinia IgG MBCs persist for up to 50 years of vaccination with vaccinia 27. Similarly, HBV‐specific MBCs persist after hepatitis B vaccination 28, despite the fact that half of the vaccines lose the cognate Abs within a few years of vaccination 32. There are also similar observations in HIV where IgG MBCs specific for the conserved neutralizing CD4 induced or CD4‐binding site epitopes of gp120 are maintained in the absence of their cognate serum Abs 33. In the case of malaria, rapid boosting of several antimalarial Ab responses has been reported in both children and adults after reexposure to parasites following prolonged periods of either sustained control or droughts 34, and in areas of very low transmission where malaria infections rarely overlap 36, suggesting that humans can generate and sustain *P. falciparum* specific MBC. Recent studies quantifying malaria antigen‐specific MBCs in mice 37 and in humans 38 confirmed that *Plasmodium*‐specific MBCs can live longer in some but not all exposed individuals. However, the determination of the longevity of *P. falciparum* specific MBCs is complicated by the seasonal nature of malaria transmission in endemic areas, and possible boosting by recent or chronic asymptomatic infections in areas of low transmission. For example, although Weiss et al. 40 found that *P. falciparum* specific MBCs are inefficiently acquired during the transmission season, they could not determine longevity beyond the ensuing dry season, as it was interrupted by the next transmission season. Recent studies have taken advantage of the dramatic reduction in *P. falciparum* transmission in previously endemic areas, to determine longevity of B‐cell memory 38. For example, Wipasa et al. 38 reported stable malaria‐specific Ab and MBC levels in adults with a clear history of prior exposure to malaria but living in an area of extremely low transmission in Thailand, while we reported long‐term maintenance of MBCs but not plasma Abs in children following over 5 years of interrupted *P. falciparum* exposure in Kenya 39. Such studies should be complemented by similar investigations following infection of individuals living in areas without malaria transmission. Although there are limited data on *P. falciparum* specific Abs induced from natural infections of travelers returning to malaria‐free countries 42, there are currently no data including malaria antigen‐specific MBC analysis.

The aim of our study was to determine whether natural *P. falciparum* infections induce enduring malaria‐specific IgG Abs and MBCs in returning travelers that became infected in the tropics, but subsequently lived free of malaria in Sweden. We therefore compared levels of circulating *P. falciparum* and tetanus (TT) specific IgG Abs and MBCs between travelers that were treated for malaria at the Karolinska University Hospital, Stockholm, Sweden, between 1994 and 2010, malaria‐naïve Swedes living in Stockholm, and adults who lived in an endemic area of Kenya all their lives (“immune adults”).

## Results

### Characteristic of study subjects

IgG MBC and Ab responses to TT and the malaria antigens apical merozoite antigen‐1 (AMA1) (a mixture of the two alleles of AMA1‐FVO and 3D7 in a 1:1 ratio), MSP1‐42 kDa, merozoite surface protein‐3 (MSP3), and *P. falciparum* lysate were determined in 47 Swedish residents diagnosed with *P. falciparum* following travel in tropical countries (“travelers”). Antigen‐specific IgG MBCs and Abs were quantified using ELISpot and ELISA assays. The travelers were defined as residents of Sweden who were diagnosed and treated at Karolinska University Hospital, Stockholm, with acute malaria, as identified from the hospital records and were either born in Europe (*n* = 33) or in countries known to have malaria transmission (*n* = 14) (Supporting Information Table 1). Apart from one traveler who was HIV positive, there were no other reports of immunosuppressive conditions or treatments. The median time since the acute malaria episode to the time of sampling for the travelers was 11 (range, 1–17) years. For comparison of MBC and Ab levels, we selected eight Swedish adults that were malaria naïve (“naïve controls”) and 14 malaria immune adults that had lived all their lives in Junju location, Kilifi District, Kenya, an area that is endemic for *P. falciparum* transmission. None of the immune adults were parasitemic by microscopy at the time of sampling. However, *P. falciparum* parasite prevalence was estimated at 30% among children in their location 39.

### *Plasmodium falciparum* specific IgG‐MBCs are maintained for longer periods than their cognate Abs

**Figure 1:**
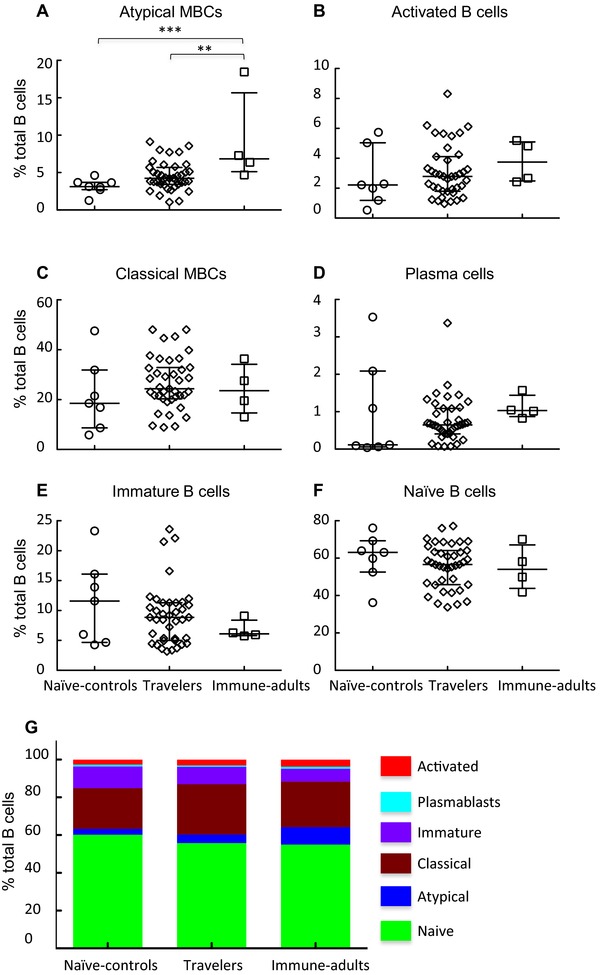
Anti‐*Plasmodium falciparum* MBCs, but not their cognate Abs, live longer. Levels of MBCs and Abs were determined from cross‐sectional samples by ELISA and ELISpot, respectively. Each sample was tested in duplicate and triplicate for ELISA and ELISpot, respectively. MBC frequencies are expressed per million of cultured PBMCs. Shown are the comparisons of B‐cell memory responses between malaria‐naïve adults (open circles), travelers (open diamonds), and immune adults (open squares) for levels of antigen‐specific IgG Abs (left) and the frequencies of antigen‐specific IgG MBCs (right). Each symbol represents an individual donor and horizontal bars indicate median ± interquartile range. Dashed lines indicate the Ab cut off, being the mean + 2 SDs of the naïve controls. Statistical significance was determined with the Kruskal–Wallis test (with Dunn's correction for multiple comparisons). *p < 0.05; **p < 0.01; ***p < 0.001.

Apart from the *P. falciparum* lysate‐specific Ab levels, where 30% of the travelers had levels above naive controls, all the other malaria‐specific Abs were at background levels among this group (Fig. 1 and Table 1). In contrast, 59, 45, and 28% of travelers had MBC specific for AMA1, merozoite surface protein‐1 (MSP1), and MSP3 (after the subtraction of BSA‐specific background), respectively, and 37 of the 47 (78%) travelers had MBC specific for at least one of the three malaria antigens (Table 1). Collectively, these data suggest that the MBCs to these *P. falciparum* antigens are maintained for much longer than their cognate Ab.

**Table 1:** Profile of antibody and memory B cell responses to *P. falciparum* antigens and tetanus toxoid in travelers who were diagnosed and treated for malaria 1‐17 years earlier

Traveler	Country	Years in	cPost‐	Time	*P. falciparum*	MSP1‐	MSP3‐	AMA1‐	dMBC	TT‐MBC
	of birth	Sweden	malaria	since	lysate Ab	MBC	MBC	MBC	breadth	(ELISpot)
		before	travel	malaria	(ELISA)	(ELISpot)	(ELISpot)	(ELISpot)	(ELISpot)	
		travel		(years)						
15	Ghana	15	0	8	X			X	1	X
2	Sweden		0	10			X	X	2	X
42	Ivory Coast	0	0	11	X	X			1	
7	Sweden		0	13			X		1	X
13	Sweden		0	16			X	X	2	X
39	Sweden		1	1				X	1	X
40	Uganda	17	1	1	X	X		X	2	X
27	Sweden		1	2	X	X	X	X	3	X
43	Sweden		1	2					0	
47	Sweden		1	2					0	X
30	Gambia	40	1	2	X	X			1	X
22	Sweden		1	2		X		X	2	X
41	Kenya	65	1	2			X	X	2	X
48	Sweden		1	4				X	1	
17	Ghana	32	1	6	X	X	X	X	3	X
25	Sweden		1	8			X	X	2	X
1	Sweden		1	9				X	1	X
32	Sweden		1	11					0	X
12^a^	Sweden		1	11					0	X
14	Sweden		1	11		X		X	2	X
6	Sweden		1	11		X	X	X	3	X
28	Ghana	12	1	12	X	X		X	2	X
21	Sweden		1	12	X	X	X	X	3	X
9	Gambia	21	1	12		X	X	X	3	X
11	Sweden		1	13					0	X
18	Netherlands		1	13	X	X	X	X	3	X
19	Sweden		1	13		X	X	X	3	X
35	Gambia	4	1	14					0	X
36	Gambia	21	1	14	X			X	1	X
24	Sweden		1	15			X	X	2	X
8	Sweden		1	15					0	X
3	India	6	1	15					0	X
31	Sweden		1	15				X	1	
37	Sweden		1	15	X	X			1	X
29	Sweden		1	15	X	X	X	X	3	X
4	Uganda	11	1	16		nd	nd	nd	nd	
16	Sweden		1	16	X	X		X	2	X
26	Sweden		1	16		X	X	X	3	X
46^b^	Finland		1	2					0	Missing data
23^b^	Sweden		1	3		X		X	2	X
33^b^	Nigeria	29	1	12	X				0	X
10	Sweden		2	4	X	X		X	2	X
34	Sweden		2	7		X			1	
5	Sweden		2	11				X	1	X
20	Eritrea	0	2	14	X	X		X	2	X
44	Sweden		2	17		X			1	
45^b^	Sweden		2	9					0	

a HIV sero‐reactive individual.

b Individuals who had self‐reported malaria episodes after their malaria diagnosis.

c Data are sorted according to the number of trips made in the intervening period between malaria treatment in Sweden and sampling. Clear rows represents the five travelers that did not travel back to countries known to have malaria (including countries with any level of transmission).

d Number of *P. falciparum* antigens that an individual had MBC for.

The light and dark shades of grey represents travelers that either made quick short trips, or lived for more than year in endemic countries, respectively.

X indicates a positive response to the respective antigen.

Similarly, the proportions of Kenyan immune adults that were positive for *P. falciparum* antigen‐specific Abs were consistently lower than those positive for MBCs (40 versus 79%, 0 versus 85.7%, 38 versus 64.2% for AMA1, MSP1, and MSP3, respectively).

We identified 17 travelers that were unlikely to have been reexopsed to malaria during the intervening period between treatment of the index episode in Sweden and this study. Twelve of these traveled to countries with extremely low transmission in Asia, while five (travelers 15, 2, 42, 7, and 13) did not travel at all (Table 1). Although all the 17 had maintained *P. falciparum* specific MBC, these five that did not travel back provides the stronges tevidence for long‐term maintenance of MBCs in absence of reexposure to parasites. Each of them had maintained MBCs specific for at least one of three *P. falciparum* antigens tested for a median of 12 years (range 8–16) since the diagnosis and treatment of the index episode, confirming that humans can generate and maintain long‐lived *P. falciparum* specific MBCs in the complete absence of reexposure to parasites.

Interestingly, only one of the 11 travelers with no MBCs to any of the *P. falciparum* antigens had Abs to *P. falciparum* lysate (Table 2). In contrast, five of the 13 (38.5%) with MBCs to at least one *P. falciparum* antigen, five of the 13 (38.5%) with MBCs to at least two *P. falciparum* antigens, and five of the nine (55.6%) with MBCs to all three *P. falciparum* antigens had Abs to *P. falciparum* lysate, respectively.

**Table 2:** Breadth of *P. falciparum*‐specific ELISpot responses (i.e. number of antigens to which memory B cell responses were detected) among travelers in relation to origin and time since diagnosis

Breadth of	N (% of 47)	Born in	Time since	*P. f*‐lysate	TT MBC
*Pf*‐specific		endemic	malaria diagnosis,	Ab positive	positive
MBC		country	years median	N (%)	N (%)
responses		N (%)	(IQ range)		
0	11 (23.4)	3 (27)	11 (2‒14)	1 (9.1)	8 (72.7)
1	13 (27.6)	4 (30)	11 (7‒14)	5 (38.5)	8 (61.5)
2	13 (27.6)	3 (23)	11 (3‒14)	5 (38.5)	13 (100)
3	9 (19.1)	2 (22)	12 (11‒13)	5 (55.6)	13 (100)
ND^a^	1 (2.1)	1			

a Data is unavailable for one donor (ND), for whom we did not obtain enough cells for ELISpot experiments.

In multivariable linear regression analysis, none of the *P. falciparum* specific MBC responses were associated with the time since malaria diagnosis, being born in an endemic country, parasitemia at diagnosis, and previous episodes (Table 3). Age at the time of diagnosis with malaria was weakly but positively associated with levels of anti‐MSP3 (but not AMA1 and MSP1) MBC. The number of days with symptoms befroe treatment was weakly associated with frequencies of anti‐AMA1 and MSP1 MBC. Being born in an endemic country was positively associated with anti‐*P. falciparum* lysate Ab levels (but not with MBC responses).

**Table 3:** Multivariable analysis of associations between age at episode, time since episode, country of origin, prior malaria‐exposure, parasitaemia and status duration of symptoms with frequencies of *P. falciparum* specific MBC^a^

	AMA1 MBC (ELISpot)		MSP1 MBC (ELISpot)		MSP3 MBC (ELISpot)		Pf‐lysate Ab (ELISA)	
Covariate	Coefficient	*P*	Coefficient	*P*	Coefficient	*P*	Coefficient	*P*
	(95% CI)		(95% CI)		(95% CI)		(95% CI)	
Age at episode (yrs)	0.02 (−0.03–0.07)	0.45	−0.02 (−0.06–0.03)	0.46	0.041 (0.01–0.07)	0.03	0.01 (−0.04–0.05)	0.72
Time since diagnosis (yrs)	0.11 (−0.032–0.26)	0.12	−0.01 (−0.13–0.10)	0.82	0.10 (−0.01–0.21)	0.06	−0.02 (−0.15–0.11)	0.73
Country of origin	−0.35 (−1.67–0.97)	0.59	0.43 (−0.64–1.51)	0.41	−0.30 (−1.25–0.66)	0.53	1.70 (0.48–2.91)	0.01
Previous episode	0.20 (−1.33–1.73)	0.79	0.14 (−1.11–1.38)	0.82	−1.07 (−2.18–0.03)	0.06	−0.23 (−1.58–1.12)	0.73
Parasitaemia	−0.08 (−0.19–0.04)	0.55	−0.04 (−0.13–.06)	0.43	−.027 (−0.11–0.05)	0.50	0.03 (−0.08–0.13)	0.59
Duration of symptoms	0.19 (−0.01–0.39)	0.05	0.18 (0.02–0.34)	0.03	0.07 (−0.07–0.21)	0.30	0.04 (−0.14–0.22)	0.67

a Data for each of the four responses listed were analysed by multivariable linear regression.

The levels of anti‐TT Abs were similar across the three groups (Fig. 1). However, the immune adults had significantly higher frequencies of anti‐TT MBC, perhaps reflecting the fact that 13 of them were women of child‐bearing age, who normally receive additional booster vaccinations during antenatal clinic visits in this population. There were no differences in the frequencies of total IgG MBCs in the three groups (Supporting Information Fig. 1).

### Atypical MBC frequencies among the travelers were similar to malaria‐naïve levels

**Figure 2:**
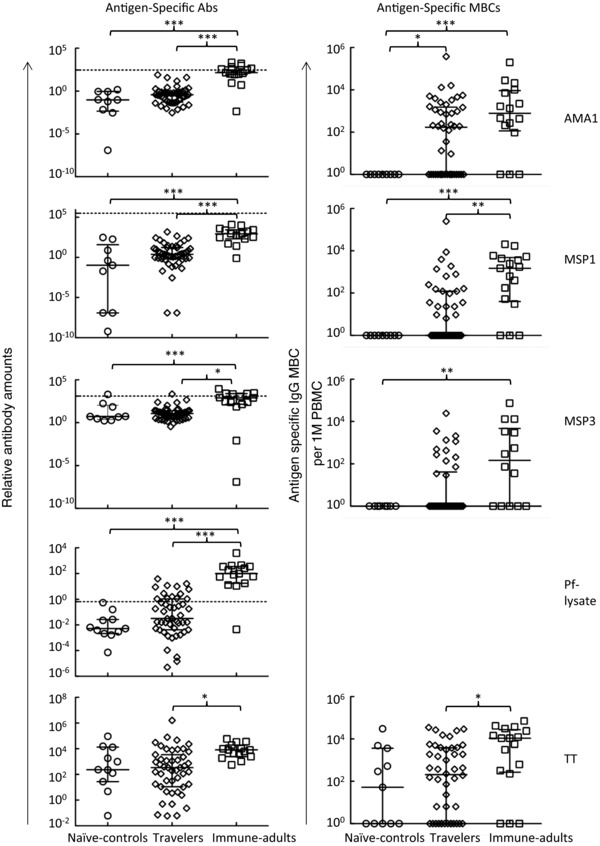
Gating strategy for flow cytometric phenotyping of B cells. Total B cells were identified by CD19 expression (top left) and then subsets were identified by the expression of CD10, CD20, CD21, and CD27. All numbers represent the percentage of the parent gate.

We next determined whether exposure to *P. falciparum* infection followed by subsequent prolonged periods of nonexposure to malaria antigen resulted in the expansions and maintenance of these atypical MBCs as well as other B‐cell subsets by comparing their relative proportions in the peripheral bloods of naive controls, travelers, and immune adults. B‐cell subsets were defined as described elsewhere 18: naïve B cells, CD19^+^CD27^−^CD21^+^CD10^−^; plasma cells, CD19^+^CD27^+^CD21^−^CD20^−^; immature B cells, CD19^+^CD10^+^; classical MBC, CD19^+^CD27^+^CD21^+^CD10‐; atypical MBC, CD19^+^CD27^−^CD21^−^CD10^−^; and activated MBC, CD19^+^CD27^+^CD21^−^CD20^+^CD10^−^. Figure 2 illustrates the flow cytometric gating strategies used in the determination of the relative proportions of B‐cell subsets and a representative comparison of the naive controls, travelers, and immune adults.

**Figure 3:**
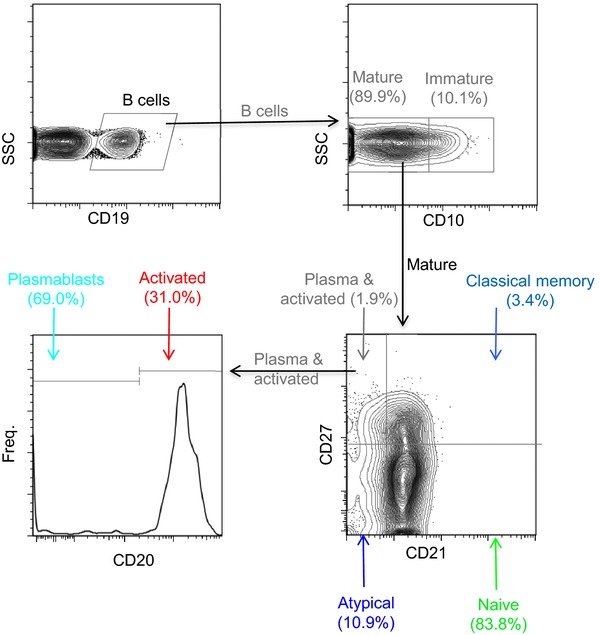
Atypical MBCs are not significantly expanded in malaria‐exposed travelers relative to naïve‐control adults. (A–F) Comparison of the proportions of (A) atypical MBCs, (B) activated B cells, (C) classical MBCs, (D) plasma cells, (E) immature B cells, and (F) naive B cells within the total CD19^+^ B‐cell compartment (as defined in Fig. 2) between the different study groups. Each symbol represents a single individual, and the solid horizontal lines indicate the median and interquartile‐range values for the respective groups. Statistical significance between various groups was determined with Kruskall–Wallis test. *p = 0.05; **p = 0.01; ***p = 0.001. (G) The relative percentages of the various B‐cell subsets out of the total CD19^+^ B cells for each of the study groups are shown.

The immune adults had an expanded atypical MBC population relative to the naive controls and the travelers. There were, however, no differences in the proportions of atypical MBCs between the travelers and the naive controls (Fig. 3A). Similarly, there were no differences in the proportions of atypical MBCs between the immigrant and Swedish‐born travelers (data not shown).

There were no significant differences between the travelers and either of the naive controls or immune adults for activated B cells, classical MBCs, plasma cells, immature and naïve B cells (Fig. 3B–F, respectively). Similarly, there were no differences in the proportions of activated B cells, classical MBCs, plasma cells, immature and naïve B cells between the immigrant, and Swedish‐born travelers (data not shown).

## Discussion

We found that travelers returning to Sweden with acute malaria generated and maintained *P. falciparum* specific MBCs for up to 16 years in the complete absence of reexposure to malaria parasites. These MBCs were largely maintained in the absence of their cognate‐specific Abs in contemporaneous plasma, suggesting that they are the longer lived of the two *P. falciparum* specific B‐cell memory compartments (i.e. Ab‐producing plasma cells versus MBCs). In addition, we found similar frequencies of atypical MBC between exposed travelers and naïve controls, confirming that persistent and/or chronic *P. falciparum* exposure is required for their maintenance 18. However, atypical MBCs were significantly expanded in the malaria‐immune adults relative to both the travelers and naïve controls.

Previous studies have suggested that malaria infections may not induce long‐lived B‐cell memory responses to *P. falciparum* antigens, especially in young children (reviewed in 1). Therefore, there have been efforts to determine the longevity of *Plasmodium* antigen‐specific B‐cell memory in animal models 37 and in human studies 38. Because of possible confounding by boosting from recent reinfections, such studies are only possible in areas with very low *P. falciparum* transmission, or in people living in malaria‐free countries that were either exposed to malaria experimentally or naturally during travel in endemic areas 42. Such studies have demonstrated long‐lived cellular (cytokine) responses to *P. falciparum* and limited Ab responses 49. Our data demonstrate that *P. falciparum* specific MBCs may live longer despite absent Ab production.

Although 17 of the travelers had only traveled back to countries with extremely low transmission in Asia and had maintained long‐lived *P. falciparum* specific MBC, the strongest evidence for maintenance in absence of reexposure came from the five that did not travel to any malaria‐endemic area. These five travelers had maintained *P. falciparum* specific MBCs to at least one of three *P. falciparum* antigens tested for between eight and 16 years without any evidence of reexposure. These findings of long‐lived *P. falciparum* specific IgG MBCs (for up to 16 years, the longest follow‐up time in this current study) in the complete absence of persisting exposure are reassuring in the face of reports of declining *P. falciparum* transmission in Africa 50. Thus, these data suggest that immunological memory to *P. falciparum* antigens is not as short‐lived as previously presumed. Nonetheless, future studies should investigate why not all the travelers in the current study, or the individuals in previous studies 38 were positive for *P. falciparum* specific MBC, in spite of clear evidence for prior exposure.

The finding of long‐lived *P. falciparum* specific MBCs in the absence of reexposure is consistent with a previous report suggesting that naturally acquired immunity to malaria could persist for several years of nonexposure 48. Although MBCs may have many different functions, their most direct contribution to immunity against infection is through production and hence maintenance of protective levels of preexisting Ab. Alternatively, because the stimulation threshold for subsequent proliferation and differentiation of MBC into Ab‐secreting cells is low, MBCs can contribute to the rapid redeployment of protective Ab levels, upon reinfection. However, as argued previously 39, the rate of proliferation and differentiation of MBCs into Ab‐secreting cells must overtake the pathogen's replication rate and development of associated pathogenesis. Because the expression of some merozoite‐stage antigens begins during liver stage development of *P. falciparum* 53, there is a possibility that proliferation and differentiation of malaria blood‐stage‐specific MBCs into Ab‐secreting cells is initiated early enough in the infection, ensuring that the newly induced Ab checks parasite replication and hence protects the host from pathology. However, this possible function of *P. falciparum* specific MBCs is yet to be demonstrated in prospective studies. Although we reported a lack of prospective association with protection for anti‐MSP1 and AMA1 IgG MBCs in a recent study 20, the number of children included in that analysis was small. Sufficiently powered future studies should investigate whether the specific Ab that would be rapidly deployed by long‐lived MBC, upon reinfection of the immune individual are protective against clinical malaria.

In summary, we conclude that although persistent exposure to malaria antigen is required for the maintenance of circulating *P. falciparum* specific Abs in endemic areas, *P. falciparum* specific MBCs can be maintained independently of sustained exposure to the parasite. This finding has major implications on future immunoepidemiological studies. Such studies would gain more information on immunological memory, or previous antigenic exposures by combining serological analysis of antigen‐specific Abs with quantification of their cognate MBCs. Otherwise, studies investigating humoral responses to *P. falciparum* antigens in areas of declining *P. falciparum* transmission by serology alone would greatly underrepresent past *P. falciparum* specific Ab responses. Having shown that *P. falciparum* specific MBCs can be long‐lived in the absence of reexposure (in agreement with recent studies), future studies should now investigate the possible protective role for *P. falciparum* specific MBCs in prospective studies of individuals exposed to endemic malaria. Such studies will help identify protective long‐lived IgG MBC specificities that can then be targeted for induction by vaccination.

## Materials and methods

### Ethics

Ethical approval was obtained from the Central Ethical Review Board, Stockholm, and the Kenyan Medical Research Institute (KEMRI) National Ethics Committee. Written informed consent was subsequently obtained from the respective participants.

### Study sites

The study was done at the Karolinska University Hospital in Stockholm. The hospital serves ∼ 2 million people living in Stockholm. The Department of Infectious Diseases treats up to 50 cases of malaria in returning travelers per year. In addition, samples from malaria immune adults were obtained from the KEMRI, Centre for Geographic Medicine Research Coast situated at Kilifi District Hospital, Kenya. The hospital serves ∼ 240 000 people living in Kilifi District. The immune adults were life‐long residents of Junju location, which remains stably endemic with two high‑transmission seasons (May to August, and in October to December).

### Study populations

Patients admitted with a *P. falciparum* malaria episode to Karolinska University Hospital between 1994 and 2010 were invited to participate in a cross‐sectional study of malaria‐specific B‐cell memory in May 2011. An invitation letter was sent out to 270 patients along with a description and aim of the study. Out of 270 invitees, 47 individuals consented to participate and came to a visit at the hospital for blood sampling and to fill out a questionnaire. Additionally, for comparison we recruited eight malaria‐naïve Swedish born adults and 14 malaria immune adults from the Kenyan coast (where cross‐sectional parasite prevalence amongst children is 30% 54).

### Questionnaire

Demographic data in the questionnaire included; age, sex, nationality, country of origin, country visited prior to the *P. falciparum* infection, and prophylaxis use. Travel history data was essential for identifying possible exposure to *P. falciparum* infections. The participants also filled visits to malaria‐endemic countries and self‐reported malaria episodes prior to and after admission at Karolinska University Hospital into the questionnaire. Clinical data from the malaria episode was extracted from clinical records including symptoms, severity of disease, and parasitemia parameters. Demographic information was included to allow for comparisons between Swedish born and immigrants from malaria‐endemic areas.

### Peripheral blood mononuclear cells (PBMCs) and plasma

Thirty milliliters of venous blood was collected in heparinized tubes for PBMC isolation and 10 mL in EDTA tubes for plasma and DNA extraction. The PBMCs were isolated by density centrifugation over Ficoll‐Paque. PBMCs and plasma for ELISpot and ELISA were harvested and stored in liquid nitrogen and −80°C, respectively.

### Antigens

*Plasmodium falciparum* specific IgG MBC and serum Ab responses were quantified against recombinant *P. falciparum* AMA1 (a mixture of the two alleles of AMA1‐FVO and 3D7 in a 1:1 ratio), MSP1 42 kDa, and merozoite surface protein‐3 (MSP3) to which circulating IgG Abs have been associated with clinical protection in previous studies 56. The respective recombinant *P. falciparum* antigens were provided by Dr. Louis Miller (NIH, USA), while *P. falciparum* lysate, was made from the ITO parent strain sonicated on ice 60. TT was obtained from The National Institute for Biological Standards and Control (UK).

### Enzyme‐linked immunosorbent assay (ELISA)

Plasma samples were tested in duplicates for human IgG Abs specific for *P. falciparum* and TT antigens using a standard ELISA protocol, as described elsewhere 39. For AMA1, ELISA plates were coated with a 1:1 mixture of FVO and 3D7 alleles. Plates were coated overnight at 4°C, with recombinant proteins and TT at 1 μg/mL, and *P. falciparum* lysate and the accompanying red blood cell control lysate at 1 in 500 dilution in bicarbonate buffer (100 μL/well). A 100 μL/well of 1 in 1000 dilution of test plasma in 0.3% phosphate‐buffered saline (PBST) +EDTA was added after plates had been washed three times with 0.05% Tween in PBST, and thereafter blocked with 10% fetal calf serum (FCS)/PBS (200 μL/well). Plates with test plasma were then incubated for 1.5 h at room temperature in a humidified chamber and then washed five times before the addition of alkaline phosphatase (AP) labeled goat anti‐human IgG Ab (Sigma) conjugate at 1:2000 dilution 0.05% PBST at 100 μL/well. After 1‐h incubation with the conjugate, the plates were washed five times and the human IgG complexed with the AP labeled conjugate revealed with *p*‐nitrophenyl phosphate (Sigma). The substrate reaction was stopped with 50 μL per well of 3M NaOH, after which the plates were left for 5 min in the dark before being read at 405/570 nm. Purified hyperimmune IgG was used as a standard for the *P. falciparum* specific ELISAs. Anti‐TT IgG Abs were quantified against hyperimmune plasma from an adult, who received booster immunizations with the TT vaccine prior to this study. Ab concentrations were expressed in arbitrary units determined against the respective standard curves on each plate.

### Enzyme‐linked immunospot (ELISpot)

*Plasmodium falciparum* specific MBCs were quantified by a recently optimized ELISpot assay 61 as described elsewhere 39. Briefly, PBMCs were thawed and cultured for 5 days at 1 × 10^6^cells/mL of RPMI complete media with 2.5 μg/mL CpG oligodeoxynucleotide‐2006 (Eurofins MWG/Operon); 1/10 000 dilution SAC, 1/100 000 dilution pokeweed mitogen (Sigma‐Aldrich); and 50 ng/mL of IL10 (R&D Systems) in 24‐well plates. The 96‐well ELISpot plates (Millipore Multiscreen‐HA) were precoated by incubating plates overnight at 4°C with either: 10 μg/mL polyclonal goat Ab specific for human IgG (Caltag) to detect all IgG‐secreting cells; 1% BSA as a nonspecific protein control or 5 μg/mL of tetanus toxoid (TT), MSP1 or AMA1 in PBS. Plates were blocked by incubation with a solution of 10% FCS in RPMI for 2 h at 37°C. PBMCs from 5‐day cultures were serially diluted by a factor of a ½ in duplicates or triplicates (depending on the numbers of cultured PBMC available) to final concentrations of between 0.125 × 10^4^ and 1 × 10^4^ PBMC/well to detect total IgG+ ASCs, and between 0.062 × 10^5^ and 2 × 10^5^ PBMC per well to detect antigen‐specific ASCs. ELISpot plates were kept at 37°C in a 5% CO_2_ incubator for 5 h and then washed four times with PBS and four times with PBS‐0.05% Tween‐20. AP‐conjugated goat anti‐human IgG Fc Ab (Jackson ImmunoResearch Laboratories) diluted 1:1000 in PBS‐0.05% Tween‐20 with 1% FCS was added to wells and incubated overnight at 4°C. Plates were washed four times with PBS‐0.05% Tween‐20, four times with PBS, and three times with distilled water before adding BCIP/NBT 100 μL per well (Bio‐Rad). The plates were dried in the dark and spots scanned with the ImmunoSpot series 4 analyzer (Cellular Technologies LTD (CTL), Germany) and results analyzed using Immunospot version 5 software (CTL, Germany). We determined the threshold criteria for positivity at eight spots per 1 × 10^6^ PBMCs, based on the upper range of the frequency obtained for 1% BSA coating.

### Flow cytometry

Briefly, PBMCs were stained with a panel of monoclonal Abs as described elsewhere 18. The Ab panel consisted of anti‐CD10‐allophycocyanin (BD Biosciences, catalog number 332777), anti–CD20‐allophycocyanin.H7 (BD Biosciences, catalog number 641396), anti–CD19‐PerCP.Cy5.5 (eBioscience, catalog number 45–0198–42), anti–CD21‐PE (eBioscience, catalog number 12–0219–42), and anti‐CD27‐PE.Cy7 (eBioscience, catalog number 25–0279–42). Flow cytometry was performed on the Beckman Coulter CyAn ADP, and data analysis was done using FlowJo software (Tree Star).

### Statistical analyses

Log transformed Ab and MBC data were analyzed using Stata version 11 (Stata Corp, USA) and GraphPad Prism for Macintosh version 5.01 (GraphPad Software, USA). Multivariable linear regression analysis was used to test the predictive value for origin of the travelers (defined by birth in either countries where malaria is endemic or nonendemic), time since malaria diagnosis, age at admission with malaria, parasitemia at diagnosis, previous episodes, and duration of symptoms before curation. Kruskal–Wallis test (with Dunn's correction for multiple comparisons) was used to compare continuous variables between groups. For all tests, two‐tailed *p*‐values were considered significant if *p* < 0.05.

## Conflict of interest

The authors declare no financial or commercial conflict of interest. This paper is published with permission from the Director of KEMRI.

**Abbreviations**
MBC: memory B cell
AMA1: apical merozoite antigen‐1
MSP1: merozoite surface protein‐1
MSP3: merozoite surface protein‐3
PBST: phospahte buffered saline/tween

## Supplementary Material

As a service to our authors and readers, this journal provides supporting information supplied by the authors. Such materials are peer reviewed and may be re‐organized for online delivery, but are not copy‐edited or typeset. Technical support issues arising from supporting information (other than missing files) should be addressed to the authors.

**Table S1**. Characteristics of study participants
**Figure S1**. Total IgG MBC frequencies in the travelers are similar to those in the naïve and immune adult controls.

Peer review correspondence
