# Supplementary material for: Long‐lived Plasmodium falciparum specific memory B cells in naturally exposed Swedish travelers
Source: Eur J Immunol. 2013 Aug 29;43(11):2919–29. doi: 10.1002/eji.201343630 (PMC4114544; doi:10.1002/eji.201343630)
Supplement: Supplementary file 1 — Table S1. Characteristics of study participants Figure S1. Total IgG MBC frequencies in the travelers are similar to those in the naïve and immune adult controls. [file eji-43-2919-s1.pdf]

Supplementary Table 1. Characteristics of study participants

	**Travelers[a] (n=47)**	**Immune adults[b] (n=14)**	**Unexposed healthy volunteers (n=8)**
Sex, n (%)<br>Male<br>Female	 29 (61.7)<br>18 (38.3)	 1 (7.2)<br>13 (92.8)	 5 (62.5)<br>3 (27.5)
Age at bleed 2011 (years), median (range)	48 (19-73)	35 (25-57)	32 (19-45)
Origin (country of birth), n (%)<br>Malaria endemic<br>Not malaria endemic	 13 (27.6)<br>34 (72.3)	 14 (100)<br>0	 0<br>8 (100)
Country of residence	Sweden	Kenya	Sweden
Malaria exposure			
Time since malaria diagnosis, median (range)	11 (1-17)	Unknown	NA
Region where individuals were infected, n (%)<br>Africa south of Sahara<br>South-East Asia<br>India	 39 (82.9)<br>5 (10.6)<br>3 (6.3)	14 (100)<br>Kenya, Junju	0
Accumulated time in endemic areas before bleed 2011 (years), median (range)	0.4 (0 – 44)	Lifelong	0
Self-reported malaria before admission, n (%)	15 (31.9)	NA	NA
Travel to malaria endemic area after admission, n (%)	42 (89.4)	NA	NA
Self-reported malaria after admission, n (%)	4 (8.5)	NA	NA
Characteristics at malaria admission			
Age at admission (years), median (range)	39 (8-68)		
Duration of symptoms before treatment (days), mean (range) [c]	2.5 (0-14)		
Parasitaemia at diagnosis, mean (%) (range)	0.5 (0.1-23)		
Severe malaria, n (%)[d]	4 (8.3)		
Treated in intensive care unit, n (%)	3 (6.2)		
Days of hospital admission, mean (range)	2.5 (0-15)		

[a]All travelers were Swedish residents.
[b]Adults living in a malaria endemic area in Kenya

[c]Including both health care and patient delay. [d]Severe malaria according to WHO criteria, taking the threshold parasitemia of 2% in non-immune individuals into account

Supplementary Figure 1

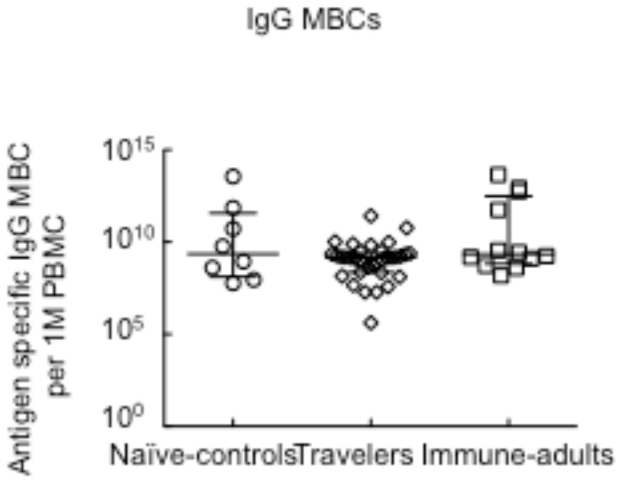

Total IgG MBC frequencies in the travelers are similar to those in the naïve and immune adult controls. Frequencies of MBC were determined from cross-sectional samples obtained in May 2011 by ELISpot, and expressed per million-cultured PBMCs. Shown are the comparisons of B-cell memory responses between malaria-naïve adults (open circles), travelers (open diamonds) and immune-adults (open squares). Horizontal bars indicate median ± interquartile range.
