# Supplementary material for: Long‐lived Plasmodium falciparum specific memory B cells in naturally exposed Swedish travelers
Source: Eur J Immunol. 2013 Aug 29;43(11):2919–29. doi: 10.1002/eji.201343630 (PMC4114544; doi:10.1002/eji.201343630)
Supplement: Supplementary file 2 — Peer review correspondence [file eji-43-2919-s2.pdf]

Peer review correspondence

European Journal of Immunology

Long-lived *Plasmodium falciparum*-specific memory B cells in naturally exposed Swedish travellers

Francis M Ndungu, Klara Lundblom, Josea Rono, Joseph Illingworth, Sara Eriksson and Anna Färnert

Corresponding author: Francis M. Ndungu, KEMRI-Centre for Geographic Medicine Research Coast

Review Timeline:	Submission date:	18 April 2013
	First editorial decision:	16 May 2013
	First revision received:	7 June 2013
	Accepted:	18 July 2013

Handling Executive Committee member: Prof. David Gray

Please note that the correspondence below does not include the standard editorial instructions regarding preparation and submission of revised manuscripts, only the scientific revisions requested and addressed.

First Editorial Decision – 16 May 2013

Dear Dr. Ndungu,

Manuscript ID eji.201343630 entitled "Long-Lived Plasmodium falciparum-Specific Memory B cells in Naturally Exposed Swedish Travelers" which you submitted to the European Journal of Immunology has been reviewed. The comments of the referees are included at the bottom of this letter.

A revised version of your manuscript that takes into account the comments of the referees will be reconsidered for publication. You should also pay close attention to the editorial comments included below.

Please note that submitting a revision of your manuscript does not guarantee eventual acceptance, and that your revision will be re-reviewed by the referees before a decision is rendered.

If the revision of the paper is expected to take more than three months, please inform the editorial office. Revisions taking longer than six months may be assessed by both the original and new referees to ensure the relevance and timeliness of the data.

European Journal of Immunology

Once again, thank you for submitting your manuscript to European Journal of Immunology and we look forward to receiving your revision.

Yours sincerely,
Karen Chu

On behalf of Prof. David Gray

Dr. Karen Chu
Editorial Office
European Journal of Immunology
e-mail: ejied@wiley.com

****

Reviewer: 1
Comments to the Author
In their concise, straightforward, and unpretentious report, Dr. Ndungu and colleagues report findings on IgG levels and functional and phenotypic B-cell parameters in individuals with very limited and long-since exposure to Plasmodium falciparum malaria parasites. They find evidence of memory B cells maintained for up to 16 years post-exposure, long after levels of parasite-specific IgG have declined to baseline.

The issue of B-cell memory to P. falciparum is currently being investigated by several laboratories following the long-standing, but not thoroughly documented, notion that immunological memory to this parasite is somehow compromised. Immunological memory in general, and to infectious diseases in particular, is of central importance to immunologists, and the topic of the manuscript is thus of direct interest to the readership of EJI.

The study is novel, as it is the first comprehensive study of long-term immunologic memory following very limited, mostly single-episode, exposure to P. falciparum antigens following naturally acquired infection.

The authors conclude that B-cell memory can be maintained for many years, and much longer than circulating antibody levels, suggesting that in the absence of regular exposure, immunologic priming may be more a more relevant marker of clinical protection to malaria than sustained IgG levels. I agree with this conclusion, although the importance of the specific antigens studied and clinical protection remains less unequivocal than the text indicates. Although several studies have found correlations between clinical protection and naturally acquired antibody responses to AMA-1, MSP-1, and MSP 3, others have not, and the relationship between P. falciparum lysate-specific responses and clinical protection is at best very

European Journal of Immunology

doubtful. It would therefore seem prudent to include text regarding this limitation of the study. In particular, the sentence on p. 16 that begins “Thus, as P. falciparum transmission falls…” needs tempering (i.e., “we now know” seems to be an overstatement).

The references and discussion of the current literature is generally competent and adequate. However, the text on the relationship between clinical protection following natural parasite exposure and antibody responses to specific P. falciparum antigens is somewhat superficial, and gives the impression of a situation better understood and less complex than is really the case.

The title of the manuscript is precise and its length consistent with the amount of data being presented.

Minor comments:
There are no indications of number of replicates in individual assays or of the number of times the experiments were repeated, although these numbers are both informative and required by the journal. This should be corrected.

In Fig. 1, interquartile ranges appear to be shown in only some panels, although this may be an artifact caused by the low resolution of the figures provided. In any case, indications of data dispersion should be shown in all panels; personally, I would suggest 95% confidence intervals rather than interquartile ranges.

Much of the very detailed information provided in Tables I and II is not of key importance, and these tables might be relegated to Supplementary Information.

Reviewer: 2
Comments to the Author
Given that the most advanced malaria vaccine, only protected ~50% of children in Phase 3 trials highlights that there is a problem with long-term immune responses against the malarial parasite in the field. Since most of the vaccines trialled have been based on antibody-mediated protection, these responses need to be investigated. There is ample evidence that memory B cells responses are generated following malarial infections but their maintenance is open to debate. Thus, any study on memory B cells in relation to malaria is significant, but must be carefully interpreted in order to rationally design the next generation of malaria vaccines. Ndungu and colleagues have investigated memory B cells responses in travellers in Sweden, previously infected with malaria.

There are some issues that need to be addressed.

1) The introduction should be a balanced view of what is known.

European Journal of Immunology

For example, the authors state “MBC being long-lived in mice (37, 38) and humans (39, 40) confirmed P. falciparum-specific MBC can be long-lived in some but not all exposed individuals”
This sentence needs to me modified as P. falciparum was not studied in mice.
As for the mouse studies, it should be noted that in one of those studies memory responses were measure only 45 days after infection, when it takes 35-45 days for one cycle of P. chabaudi malaria to clear. In the second study, memory B-cell responses were measured after 130 days by assessing antibody secreting cells after secondary challenge. No MBC response was seen 5 days after secondary challenge and a small response was measured after 10 days. Memory B-cell responses should be activated within 5-7 days of challenge. Clearly, there was a problem with memory B-cell activation.

In an another example, the authors state that “ even though Weiss et al found P. falciparum-specific MBC are inefficiently acquired during transmission season, they could not determine longevity beyond the ensuing dry season”. This study found that malaria infections did generate good memory B-cell responses but most were lost with on-going infections, within 6 months. There were very few memory B cells left to measure beyond the ensuing dry season.

2) The ELISA assay is not sensitive or the differences are hidden by the scales on Y-axes. Except for the Pf lysate, most of the immune adults and travellers had antibody titres (against 3 parasite antigens) that appear within the mean+/- 2SD and thus not significantly different. P values are not shown. Thus, no conclusion can be drawn that antibody titres are not maintained. Figure 1 shows no error bars for antibody responses in travellers or immune adults while they are shown for naive individuals.

3) In the MBC analysis, while only 21-35% of immune adults had no MBCs specific for parasite antigens AMA1, MSP1 and MSP3, 41-74% of travellers failed to maintain their MBC numbers for at least one antigen and 23% had no MBC. The error bars showing inter-quartile range overlap, suggesting that MBCs are maintained. This analysis does not take into account the significant number of individuals who did not have MBCs for that antigen. Mean with SEM would be a better test and p values need to be shown given this is the crux of the study.

4) No legend or mention was found for Figure 4, with total IgG MBCs.

5) The discussion concludes that the persistence of parasite-specific MBC is re-assuring in the face of reports of declining transmission. However, there is no evidence that these MBC will protect against infection. In fact, field studies show (reference 44) that on-going infections cause a loss of MBCs.

European Journal of Immunology

Peer review correspondence

First Revision – authors' response – 7 June 2013

Reviewer: 1

1. The authors conclude that B-cell memory can be maintained for many years, and much longer than circulating antibody levels, suggesting that in the absence of regular exposure, immunologic priming may be more a more relevant marker of clinical protection to malaria than sustained IgG levels. I agree with this conclusion, although the importance of the specific antigens studied and clinical protection remains less unequivocal than the text indicates. Although several studies have found correlations between clinical protection and naturally acquired antibody responses to AMA-1, MSP-1, and MSP 3, others have not, and the relationship between P. falciparum lysate-specific responses and clinical protection is at best very doubtful. It would therefore seem prudent to include text regarding this limitation of the study. In particular, the sentence on p. 16 that begins "Thus, as P. falciparum transmission falls…" needs tempering (i.e., "we now know" seems to be an overstatement).

□ We agree with the reviewer that even though we show that these memory B cells (MBC) are long lasting in some people, we don't know as yet they are protective. Importantly, we have used antigens for which previous studies testing associations between circulating levels and protection have produced conflicting results. Nonetheless, future studies will test whether pre-existing MBC to these antigens and others are protective. To address this limitation, we have also replaced the sentence cited by the reviewer and inserted a new sentence at in page 16. i.e.,

o "Thus, these data suggest that immunological memory to P. falciparum antigens is not as short-lived as previously presumed." (page 16, line 9)

o "Sufficiently powered future-studies should investigate whether the specific Ab that would be rapidly deployed by long-lived MBC, upon re-infection of the immune individual, are protective against clinical malaria." (page 17, line 11)

2. The references and discussion of the current literature is generally competent and adequate. However, the text on the relationship between clinical protection following natural parasite exposure and antibody responses to specific P. falciparum antigens is somewhat superficial, and gives the impression of a situation better understood and less complex than is really the case.

□ The reviewer is right; the association between various circulating antibody specificities and clinical protection against malaria is not straight forward, even though there is a general consensus that antibodies are critical. To reflect the situation more accurately, we have modified the introduction by revising the sentence below.

o "Although, immuno-epidemiological studies conducted in different geographical locations have not produced a clear picture of which antigen targets are the most important with regards to naturally-acquired immunity to malaria, there is a general consensus that Abs are critical for protection [8-10]. However, malaria specific Ab may be …….." (page 3, line 10)

3. There are no indications of number of replicates in individual assays or of the number of times the experiments were repeated, although these numbers are both informative and required by the journal. This should be corrected.

 This information is now provided in the methods and figure legends as indicated below.
o “Plasma samples were tested in duplicates for human IgG Ab specific for P. falciparum- and TT antigens using a standard ELISA protocol, as described elsewhere [39].” (Page 8, line 4)
o “PBMCs from 5 day cultures were serially diluted by a factor of a ½ in duplicates or triplicates (depending on the numbers of cultured-PBMC available)….”.(page 9, line 10)
o “Each sample was tested in duplicates and triplicates for ELISA and ELISpot, respectively.” (Figure legend 1).

4. In Fig. 1, interquartile ranges appear to be shown in only some panels, although this may be an artifact caused by the low resolution of the figures provided. In any case, indications of data dispersion should be shown in all panels; personally, I would suggest 95% confidence intervals rather than interquartile ranges.

 Median and interquartile (IQR) ranges are now shown in all the graphs. Because these data are not normally distributed, median and IQR are preferred to mean and 95% CI as the mean is highly influenced by extreme values.
o See figure 1 (at the end of this letter)

5. Much of the very detailed information provided in Tables I and II is not of key importance, and these tables might be relegated to Supplementary Information.
 The reviewer suggests that tables and 1 and 2 could be relegated to the supplementary material section. We would like to retain table 2 (now table 1) in the main text but relegate Table 1 to supplementary information, as suggested.

Reviewer: 2
1. The introduction should be a balanced view of what is known.
For example, the authors state “MBC being long-lived in mice (37, 38) and humans (39, 40) confirmed P. falciparum-specific MBC can be long-lived in some but not all exposed individuals” This sentence needs to me modified as P. falciparum was not studied in mice.
 This sentence has now been corrected to reflect this, and reads:
o “Plasmodium-specific” and not “P. falciparum-specific” MBC ….. (page 4, line 11)

As for the mouse studies, it should be noted that in one of those studies memory responses were measure only 45 days after infection, when it takes 35-45 days for one cycle of P. chabaudi malaria to clear. In the second study, memory B-cell responses were measured after 130 days by assessing antibody-secreting cells after secondary challenge. No MBC response was seen 5 days after secondary challenge and a small response was measured after 10 days. Memory B-cell responses should be activated within 5-7 days of challenge. Clearly, there was a problem with memory B-cell activation.

 We have now dropped reference 37. However, we have retained reference 38 (Ndungu, FM et al PLoS Pathog 2009. 5: e1000690) as it demonstrated that that both MSP119- specific IgG MBC and ASC are generated, and maintained above naıve background levels for over 8 months (which is a very long time in the life of a mouse!), and importantly that these long-lived MSP1-specific MBC and ASC are maintained independently of the presence of chronic infections. As the reviewer notes, we also showed, in that study, that these long-lived MBC are functional as we could reactivate them (in vivo) 130 days after primary infection, giving rise to faster secondary ASC/ Ab classic memory responses. Furthermore, the secondary MSP119-specicifc MBC response was composed of mainly (switched) IgG isotypes, while the primary response was predominated by (non-switched) IgM ASCs.

In another example, the authors state that " even though Weiss et al found P. falciparum-specific MBC are inefficiently acquired during transmission season, they could not determine longevity beyond the ensuing dry season". This study found that malaria infections did generate good memory B-cell responses but most were lost with on-going infections, within 6 months. There were very few memory B cells left to measure beyond the ensuing dry season.

 Weiss et al (2010) showed that even though most of the newly generated malaria antigen specific MBC were lost by the end of the ensuing 6-dry month period following end of transmission, they remained higher than pre-transmission (baseline) levels. Thus we cannot know for how long the residual (remnants of the newly generated) antigen-specific MBC last as transmission resumes after the 6 months of little or no transmission.

The ELISA assay is not sensitive or the differences are hidden by the scales on Y-axes. Except for the Pf lysate, most of the immune adults and travellers had antibody titres (against 3 parasite antigens) that appear within the mean+/- 2SD and thus not significantly different. P values are not shown. Thus, no conclusion can be drawn that antibody titres are not maintained. Figure 1 shows no error bars for antibody responses in travellers or immune adults while they are shown for naive individuals.

 There are no differences in anti-malaria antibody levels between the travelers and the naïve controls (p values now included as requested, Dunn's multiple comparison test). Furthermore, these levels are still at background levels (i.e. within the mean+/- 2SD of the naïve control). We take the point from the reviewer that ELISA may not be as highly sensitive as earlier implied, and have hence adjusted the text to reflect this "caution" by deleting the words highly sensitive in the sentence below.

 "Antigen specific IgG MBC and Ab were quantified using ELISpot and ELISA assays." (Page 11, line 4)

3) In the MBC analysis, while only 21-35% of immune adults had no MBCs specific for parasite antigens AMA1, MSP1 and MSP3, 41-74% of travellers failed to maintain their MBC numbers for at least one

antigen and 23% had no MBC. The error bars showing inter-quartile range overlap, suggesting that MBCs are maintained. This analysis does not take into account the significant number of individuals who did not have MBCs for that antigen. Mean with SEM would be a better test and p values need to be shown given this is the crux of the study.

□ The reviewer is right that not all the travellers had malaria-antigen specific MBC (same as for TT). This finding is consistent with findings from previous studies where long-lived MBC have been reported in some but not all individuals tested (despite having been exposed to malaria in the past). It therefore appears that there is always a proportion of MBC negative individuals, irrespective of the antigen being tested. The reasons for this are not clear, as yet. Nonetheless, for those that had malaria antigen-specific MBC, they were long-lived (as shown in table 2).

o Because these data are not normally distributed, we have compared the medians (instead of means) by non-parametric methods (Kruskal Wallis test with Dunn's correction for multiple comparisons) and hence it is more appropriate to show the median and interquartile ranges. (see Figure 1, below).

o P values, from Dunn's multiple comparison tests are shown. (see Figure 1, below).

o "Statistical significance was determined with the Kruskal Wallis test (with Dunn's correction for multiple comparisons) (* P<0.05, ** P<0.01, *** P<0.001)." (See figure legend 1, page 29, line 9).

4) No legend or mention was found for Figure 4, with total IgG MBCs.

□ The figure legend was uploaded together with the supplementary information at the end of the manuscript. It will be uploaded as per instructions. (see supplementary figure 1 and its legend below, below).

5) The discussion concludes that the persistence of parasite-specific MBC is re-assuring in the face of reports of declining transmission. However, there is no evidence that these MBC will protect against infection.

□ The picture emerging from field studies is that MBC can be long-lived (see cited references below: Ndungu, FM Proc Natl Acad Sci U S A. 2012; Wipasa J, PLoS Pathog. 2010), and the current study in Swedish travellers that had minimal exposure to malaria strengthens this evidence further. Future studies will/are planned to investigate whether these MBC specificities and others are protective against malaria. We have suggested as much in the discussion.

o "Sufficiently powered future-studies should investigate whether the specific Ab that would be rapidly deployed by long-lived MBC, upon re-infection of the immune individual, are protective against clinical malaria, in the future." (page 17, line 11)

References:

1. Ndungu FM, Olotu A, Mwacharo J, Nyonda M, Apfeld J, Mramba LK, et al. Memory B cells are a more reliable archive for historical antimalarial responses than plasma antibodies in no-longer exposed children. Proc Natl Acad Sci U S A. 2012;109(21):8247-52. Epub 2012/05/09.

2. Wipasa J, Suphavilai C, Okell LC, Cook J, Corran PH, Thaikla K, et al. Long-lived antibody and B Cell memory responses to the human malaria parasites, Plasmodium falciparum and Plasmodium vivax. PLoS Pathog. 2010;6(2):e1000770. Epub 2010/02/23.
3. Ndungu FM, Cadman ET, Coulcher J, Nduati E, Couper E, Macdonald DW, et al. Functional memory B cells and long-lived plasma cells are generated after a single Plasmodium chabaudi infection in mice. PLoS Pathog. 2009;5(12):e1000690. Epub 2009/12/17.
4. Weiss GE, Traore B, Kayentao K, Ongoiba A, Doumbo S, Doumtabe D, et al. The Plasmodium falciparum-specific human memory B cell compartment expands gradually with repeated malaria infections PLoS Pathog. 2010;6(5):e1000912. Epub 2010/05/27.

Second Editorial Decision – 7 July 2013

Dear Dr. Ndungu,

It is a pleasure to provisionally accept your manuscript entitled "Long-Lived Plasmodium falciparum-Specific Memory B cells in Naturally Exposed Swedish Travelers" for publication in the European Journal of Immunology. For final acceptance, please follow the instructions below and return the requested items as soon as possible as we cannot process your manuscript further until all items (copyright forms etc.) are dealt with.

Please note that EJI articles are now published online a few days after final acceptance (see Accepted Articles: http://onlinelibrary.wiley.com/journal/10.1002/(ISSN)1521-4141/accepted). The files used for the Accepted Articles are the final files and information supplied by you in Manuscript Central. You should therefore check that all the information (including author names) is correct as changes will NOT be permitted until the proofs stage.

We look forward to hearing from you and thank you for submitting your manuscript to the European Journal of Immunology.

Yours sincerely,
Karen Chu

on behalf of Prof. David Gray

Dr. Karen Chu
Editorial Office
European Journal of Immunology
e-mail: ejied@wiley.com
